# Wearable Electrochemical Sensor for Sweat‐Based Potassium Ion and Glucose Detection in Exercise Health Monitoring

**DOI:** 10.1002/open.202300217

**Published:** 2024-03-05

**Authors:** Lei Ma, Wenhao Hou, Zhi Ji, Ziheng Sun, Muxi Li, Bolin Lian

**Affiliations:** ^1^ Department School of Information Science and Technology Nantong University 9# Seyuan road, Chongchuan district Nantong China; ^2^ Department School of Life science Nantong University 9# Seyuan road, Chongchuan district Nantong China

**Keywords:** Electrochemistry, Microfluidic technology, Sensors, Sweat analysis, Wearable devices

## Abstract

The increasing prevalence of wearable devices has sparked a growing interest in real‐time health monitoring and physiological parameter tracking. This study focuses on the development of a cost‐effective sweat analysis device, utilizing microfluidic technology and selective electrochemical electrodes for non‐invasive monitoring of glucose and potassium ions. The device, through real‐time monitoring of glucose and potassium ion levels in sweat during physical activity, issues a warning signal when reaching experimentally set thresholds (K+ concentration at 7.5 mM, glucose concentrations at 60 μM and 120 μM). This alerts users to potential dehydration and hypoglycemic conditions. Through the integration of microfluidic devices and precise electrochemical analysis techniques, the device enables accurate and real‐time monitoring of glucose and potassium ions in sweat. This advancement in wearable technology holds significant potential for personalized health management and preventive care, promoting overall well‐being, and optimizing performance during physical activities.

## Graphical abstract

This study developed a wearable device to provide users with real‐time insights into their physiological state during exercise. By collecting sweat and utilizing electrochemical techniques, it detects glucose and potassium levels and offers individuals a molecular‐level health perspective. It has the potential to aid in disease prevention and exercise motivation.

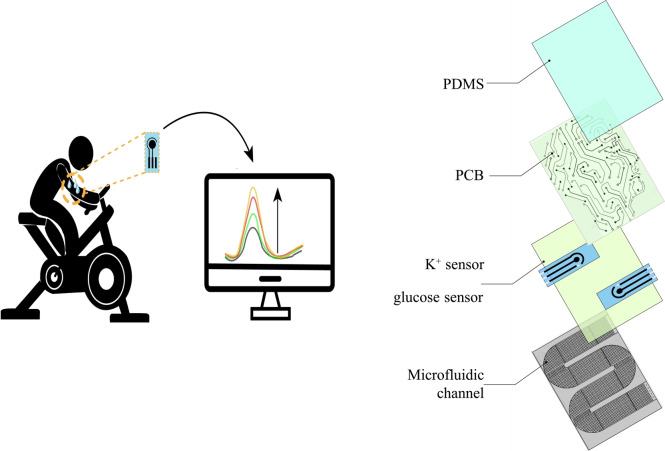

## 1 Introduction

Flexible and wearable technology is one of the rapidly developing scientific research direction in recent years. It is widely applied in fields such as human physiological activity monitoring, disease prevention, artificial skin, and human‐machine interaction.^[1]^ Considering the exponential growth of the global population, harnessing wearable technology can significantly enhance healthcare services. By enabling remote monitoring of individuals′ exercise and health, wearable technology aids in the collection and transmission of health information to healthcare professionals, effectively mitigating the strain on healthcare systems.^[2]^

Exercise health monitoring plays a crucial role in providing individuals with valuable insights into their exercise status and overall well‐being.^[3]^ However, negative physical conditions like dehydration and low blood glucose levels can significantly impact the effectiveness of exercise.^[4]^ Dehydration, for example, can cause bodily dehydration and disrupt electrolyte balance, affecting muscle contraction and nerve conduction.^[5]^ Consequently, this can result in reduced exercise capacity, muscle spasms, and increased fatigue. Similarly, low blood glucose levels can lead to insufficient energy supply to the brain, affecting cognitive function and motor coordination. Additionally, low blood glucose levels impede the muscles′ ability to metabolize glucose, resulting in diminished exercise capacity and endurance. Therefore, it is crucial to manage proper hydration and blood glucose levels during exercise and make necessary adjustments based on individual circumstances.[^6^, ^7^, ^8^]

In traditional clinical diagnostics, blood has been considered the gold standard fluid for detecting substances like blood glucose. However, invasive blood sampling procedures can instill fear and resistance in some individuals. Alternatively, sweat, as a readily available and relatively easy‐to‐obtain biofluid, holds potential as a viable alternative for daily health monitoring.^[9]^ By utilizing sweat analysis, non‐invasive monitoring of physiological parameters becomes more feasible, providing a less intrusive method for assessing health status during exercise.

However, the collection and analysis of sweat pose several challenges due to various factors. These challenges include the low sweat rates from individual sweat glands, small sample volumes, the widespread distribution of sweat glands, and uneven evaporation on the skin surface.^[10]^ These factors make the collection of sweat samples complex and prone to issues such as contamination of fresh sweat by old sweat or skin contaminants. Therefore, addressing these challenges is crucial when integrating sweat with wearable devices.

**Figure 1:**
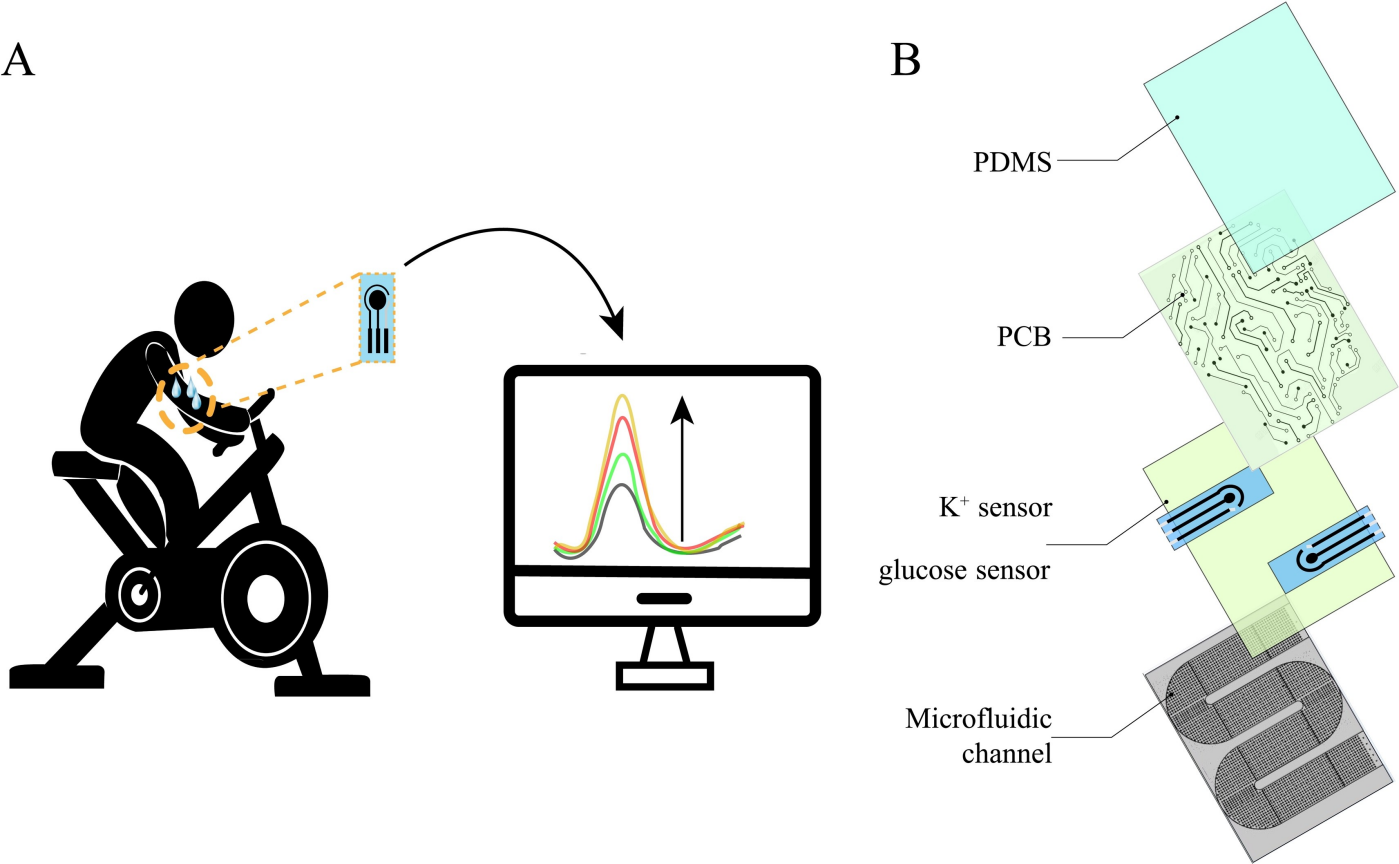
(A) Equipment usage diagram; (B) Product structure diagram.

The advancement of microfluidic technology provided possibilities for dealing with this issue by facilitating sweat collection and analysis. Microfluidic technology can effectively collect and transport small amounts of sweat from the skin for long‐term and continuous biomarker monitoring. Figure 1.A shows that we integrated a microfluidic device for sweat collection and transport to develop a device for reminding users to prevent low blood glucose and dehydration during home exercise routines. As shown in Figure 1.B, the device includes a system with a short microfluidic channel that guides sweat appearing on the skin into a small compartment within the device to form quantifiable droplets. The collected sweat droplets are then detected by integrated sensors, and the data is continuously transmitted to a computer or smartphone. After analysis, the user is promptly alerted to the impending risk of dehydration and low blood glucose levels.

The current healthcare system is moving towards connected care solutions and measuring users′ physical conditions in their home environments.^[11]^ For healthy individuals, sweat sensors have the potential to play a role in disease prevention and motivating people to exercise.^[12]^ This research primarily aims to explore the applications of sweat sensing in exercise environments, particularly with the increasing awareness of healthcare. By non‐invasively monitoring biofluids rich in biomarkers, this device offers individuals a molecular‐level perspective on health. ^[13]^ The wearable device is designed to offer users real‐time and personalized information about their physiological condition without the need for heavy equipment and medical professionals in hospitals or laboratories.

## Experimental Section

### Materials

Phosphate buffer solution (PBS) (1 M, pH=7.4), 2.5 % Glutaraldehyde glucose oxidase (GOX), BSA(≥98 %), Ethanol absolute,, Valinomycin ≥98 % (TLC), ≥95 % (HPLC), NaTPB, DOS(99 %), Cyclohexanone 0.1 mol/l were purchased from Sigma‐Aldrich. PEDOT:PSS (1.3 wt % dispersion in H2O)was purchased from MERCK, PVB was purchased from Macklin, PVC, NaCl were purchased from Shanghai Yanaye Bio‐Technology Co., Ltd

### Electrode

The ion‐selective electrode we have manufactured consists of one electrode disk and two electrode arcs. The electrode disk, with a diameter of 3 mm, is made of carbon paste and serves as the working electrode. The longer electrode arc is also made of carbon paste and serves as the auxiliary electrode. The shorter electrode arc is a silver chloride electrode and serves as the reference electrode. Refer to Figure 3.A.

As shown in Figure 3.B, when sweat is generated and collected into microfluidic channels, it sequentially passes through the glucose detection electrode and potassium ion detection electrode. When passing through the glucose detection electrode, the glucose in the sweat reacts with the glucose oxidase on the electrode, generating an electrical signal. When the sweat flows through the potassium ion detection electrode, the potassium ions in the sweat are selectively permeated through the ion‐selective membrane on the electrode, generating an electrical signal.

### Glucose electrode

**Figure 2:**
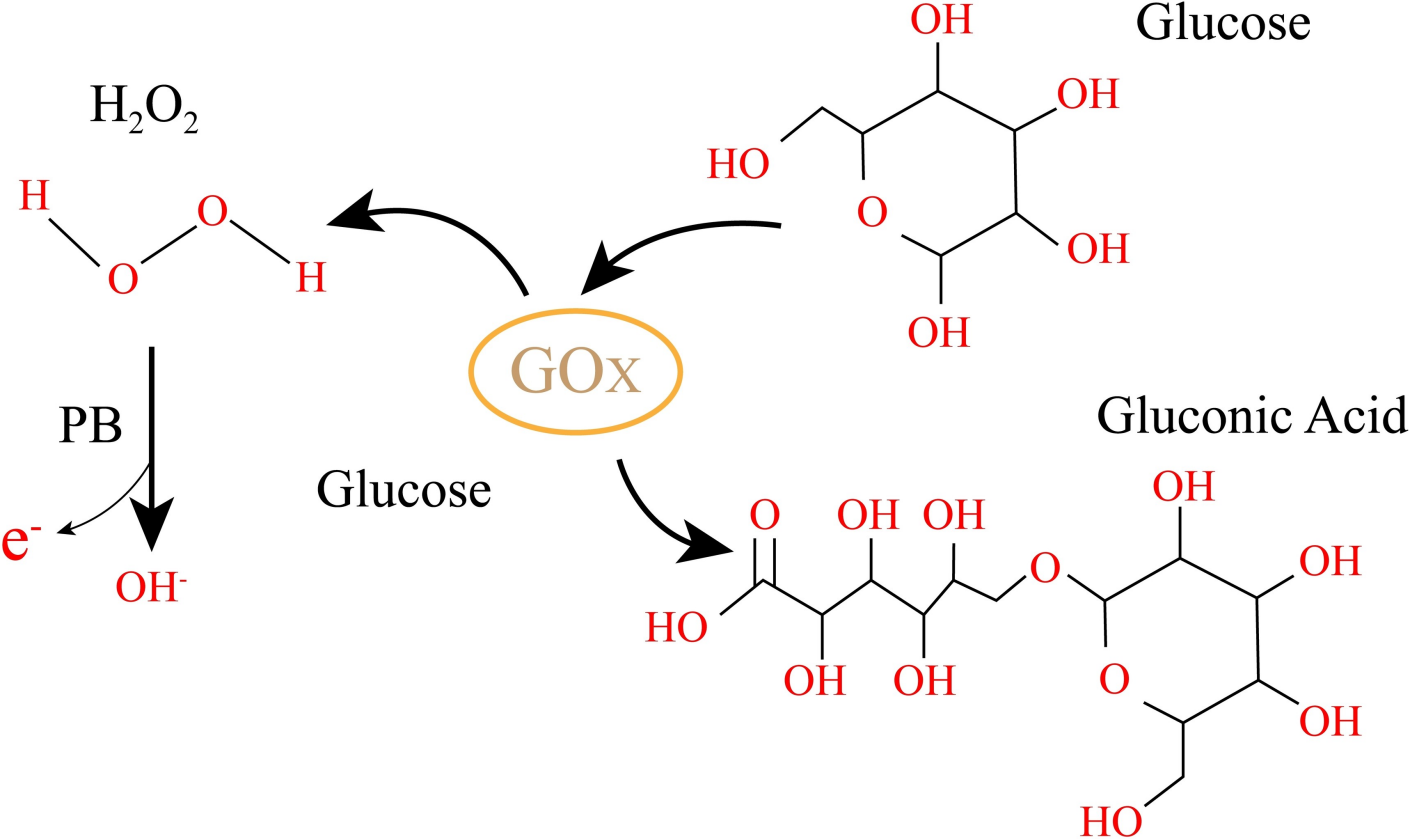
Schematic diagram of glucose detection using Gox.

In this study, we utilized a solution containing 40 mg/mL of glucose oxidase (GOx) in a volume of 2 μL to modify the working electrode for electrochemical glucose detection.^[14]^ As shown in the Figure 2. The electrode modification was carried out in a 0.1 M phosphate‐buffered saline (PBS) solution containing 10 mg/mL bovine serum albumin (BSA). The PBS buffer was used to maintain the pH of the modification solution and prevent degradation and deactivation of the GOx protein. Additionally, the PBS solution contained salts such as NaCl, which increased the ionic strength of the modification solution and enhanced the adsorption of GOx onto the electrode surface. After the modification process, the electrode was dried at room temperature to ensure the stability of the modification layer.^[15]^

Subsequently, we used 0.5 μL of a 0.5 % glutaraldehyde solution as a cross‐linking agent to immobilize GOx onto the electrode surface, forming a GOx‐modified working electrode. This step aimed to enhance the fixation and stability of GOx on the electrode surface. The cross‐linking agent and modified electrode were dried at 4 °C to facilitate the reaction between glutaraldehyde and GOx, leading to the formation of a cross‐linked structure. Additionally, drying at a low temperature reduced the evaporation of moisture and facilitated better binding of the cross‐linking agent to the electrode surface, resulting in a more stable GOx‐modified electrode.^[16]^

**Figure 3:**
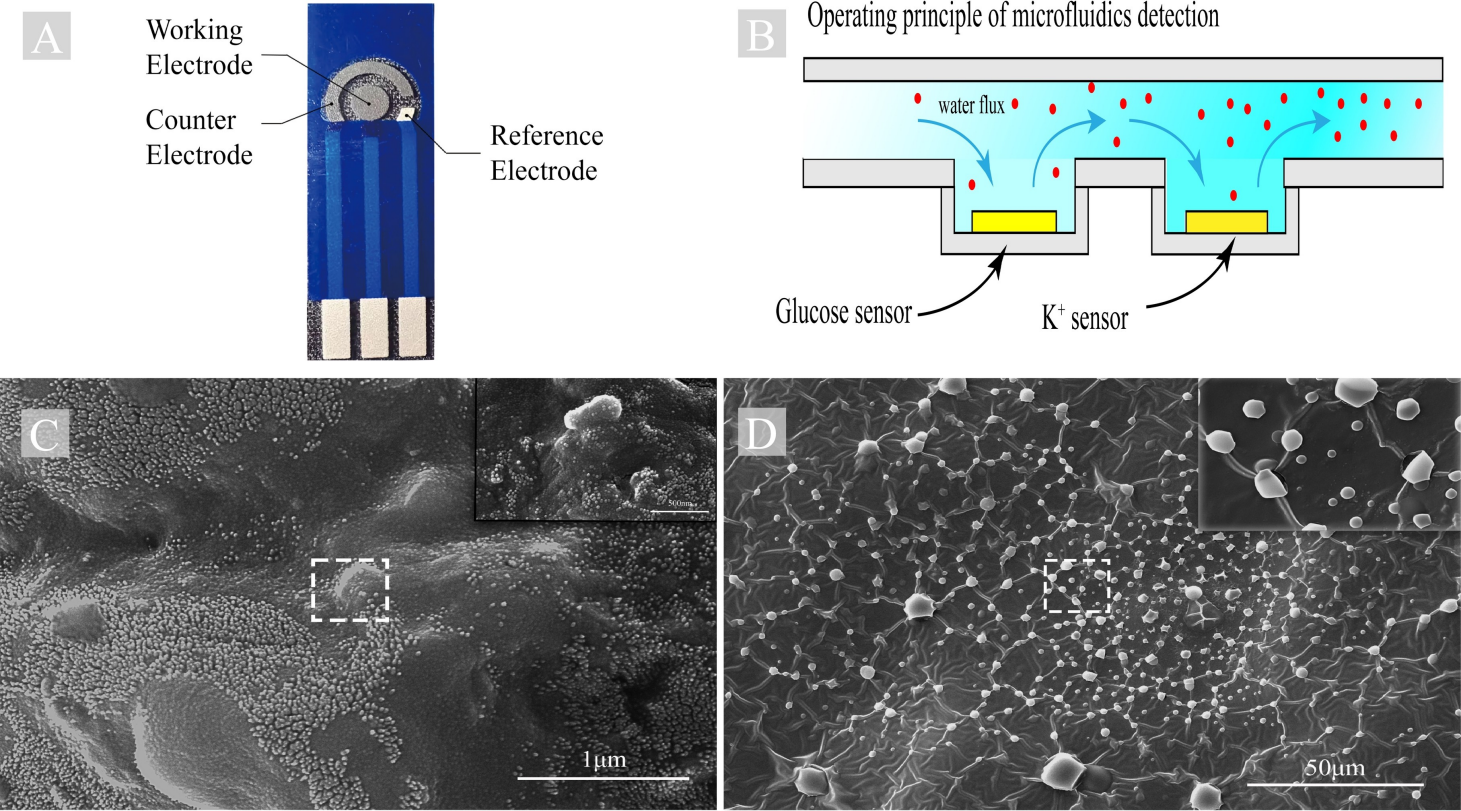
(A) Electrode before specific modification (B) Electrochemical detection in Microfluidics device (C) SEM photo of glucose electrode (D) SEM photo of K^+^ electrode.

Through the aforementioned steps, we successfully prepared a GOx‐modified working electrode with high stability and longevity, suitable for electrochemical glucose detection. As shown in the Figure 3.C.

### K^+^ electrode

In this study, we took 79.1 mg of polyvinyl alcohol (PVA) powder and 50 mg of sodium chloride (NaCl) to increase the surface area and stability of the electrode. This step aimed to adjust the ionic strength and potential in the solution, enhancing the interaction and transfer efficiency between the electrode material and ions in the solution. These substances were added to 1 mL of anhydrous ethanol and thoroughly mixed using a magnetic stirrer for 2 minutes.

Then, the obtained solution was subjected to 30 minutes of ultrasonic oscillation to ensure uniform dispersion of the components in the solution. Afterwards, we added 1 μL of the solution dropwise onto the reference electrode, repeating the process 4–8 times for a total solution volume of 4–8 μL. It is important to note that during the drop coating of the PVA solution onto the screen‐printed electrode surface, the formation of film may result in shrinkage. To avoid the situation where the center of the electrode is not covered by the PVA film, we performed multiple drops of a small amount of solution.^[17]^

Through these steps, we successfully prepared a drop‐coated PVA solution on the surface of the screen‐printed electrode, increasing the electrode‘s surface area and stability, and adjusting the ionic strength and potential. This process is expected to enhance the interaction and transfer efficiency between the electrode material and ions in the solution, providing a reliable foundation for subsequent experiments.

### Working electrode

We added 1 μL of PEDOT: PSS solution as the ion‐electron transducing medium for the ion‐selective electrode, covering the working electrode. Then, we sequentially added 2 mg of valinomycin for selective recognition and permeation of K^+^ ions, 0.5 mg of NaTPB to enhance ion selectivity and improve membrane selectivity for K^+^, and 64.7 mg of DOS (Dioctyl Sebacate) to assist in complete dissolution of PVC powder and enhance its dispersion in the solution. These components were fully dissolved in 660 μL of cyclohexanone solvent to obtain a homogeneous solution. we stirred the solution using a magnetic stirrer for 5 minutes and subjected it to 5 minutes of ultrasonic oscillation to ensure thorough and uniform mixing of the solution. After that, we dissolved 32.7 mg of PVC (polyvinyl chloride) in the solution to provide physical support and stability to the membrane. The solution was stirred using a magnetic stirrer for 30 minutes, followed by 30 minutes of ultrasonic oscillation to ensure complete dissolution of PVC. This resulted in a solution for the K^+^ selective permeation membrane. It is important to note that continuous stirring during the dissolution process is necessary, followed by ultrasonic mixing to avoid the formation of lumps with a dry exterior and wet interior in PVC.^[18]^

Finally, we added 2 μL of the respective ion‐selective membrane solution onto the surface of the working electrode and allowed it to air dry naturally.

Through these steps, we successfully prepared an ion‐selective electrode and formed the corresponding membrane layer on the surface of the working electrode to achieve selective permeation of specific ions (As shown in the Figure 3.D.).

### Microfluidic device

The microfluidic device spontaneously harvests and stores sweat. The fluid system utilizes the capillary action of sweat and the flow acceleration in the channels to guide the sweat to the sensing electrodes for electrochemical detection. Subsequently, the used sweat is timely expelled through an outlet, allowing for the chemical analysis of fresh sweat appearing on the skin without any contamination.^[19]^ The microfluidic device is primarily composed of fluid channels, analysis chambers with two electrodes, entrance section, and passageway section. The fluid channels incorporate capillary structures to accelerate the flow of the liquid. The outlet layer is made of hydrophobic materials to facilitate the smooth discharge of the analyzed sweat. We designed a microfluidic chip measuring 3.3 cm×4.4 cm×0.14 cm, with a channel depth of 0.04 cm, a collection chamber height of 0.08 cm, a width of 1.6 cm, and a length of 0.9 cm. This design can meet the requirements of high‐throughput experimental operations and ensure the accuracy and consistency of experimental data. The chip has a moderate size, suitable channel depth and collection chamber height, and is expected to achieve precise fluid control and operation.

The selection of materials is crucial in the design of microfluidic chips. We chose PMMA as the material for the chip for several reasons. Firstly, PMMA is a transparent, non‐toxic, and easy to process material, which can facilitate micro/nano processing and lithography processes. Secondly, PMMA has excellent mechanical and chemical stability, which can maintain stability and consistency under different experimental conditions. In addition, PMMA also has lower automatic fluorescence and background signals, which can reduce experimental errors and interference. Finally, PMMA has a relatively low cost and can be produced and applied on a large scale. In summary, PMMA is an ideal microfluidic chip material that can meet the needs of experimental operations and the accuracy requirements of experimental data.

### Entrance section

**Figure 4:**
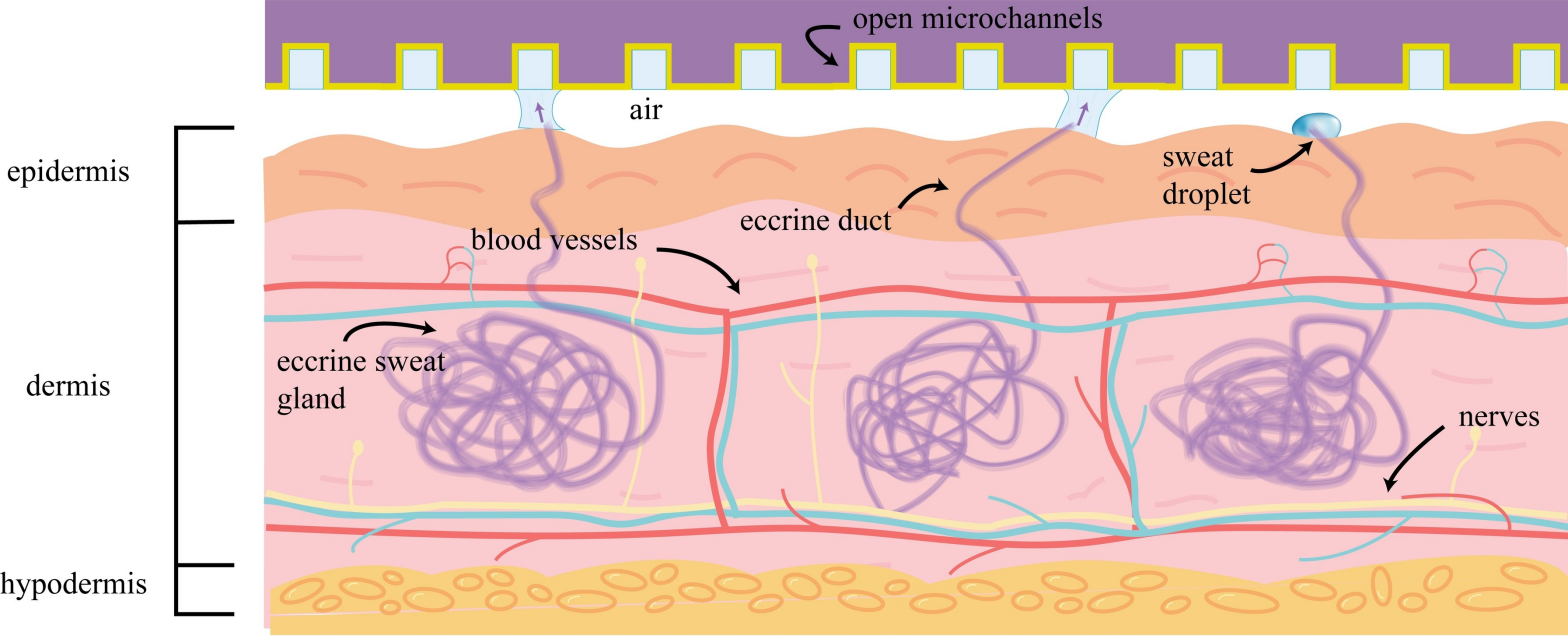
Microfluidics device absorbs sweat from skin surface.

The entrance section plays a crucial role in the microfluidic device by utilizing capillary action. Capillary action is a phenomenon where liquid is drawn into narrow channels due to the surface tension effect. As shown in the Figure 4. In the microfluidic device, efficient water uptake and stable flow processes are achieved by leveraging capillary action through appropriate geometric designs and material selection. By harnessing capillary action, the capillary inlet overcomes issues of liquid leakage and diffusion, ensuring precise sample uptake and flow control. This design approach offers flexibility and stability, providing a reliable means of sample manipulation in microfluidic experiments.^[20]^ By fine‐tuning the geometric shape and surface properties of the capillary inlet, control over different flow rates and velocities can be achieved to meet experimental requirements.

### Passageway section

In a capillary of width w and depth d, the capillary pressure can be adjusted by changing the contact angles (θ_t_, θ_b_, θ_l_, and θ_r_) between the liquid and the wall surfaces of the upper, lower, left, and right channels, respectively, according to the formula 1.


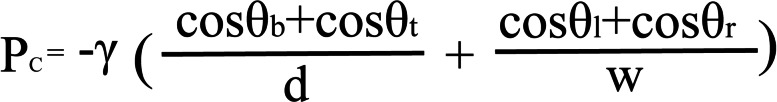




(γ is the surface tension of the liquid) When the capillary pressure in a channel segment is positive, it indicates that the segment will exert resistance to the fluid, preventing it from entering. Conversely, when the capillary pressure in a channel segment is negative, it signifies that the segment will exert attraction to the fluid, promoting its entry.

**Figure 5:**
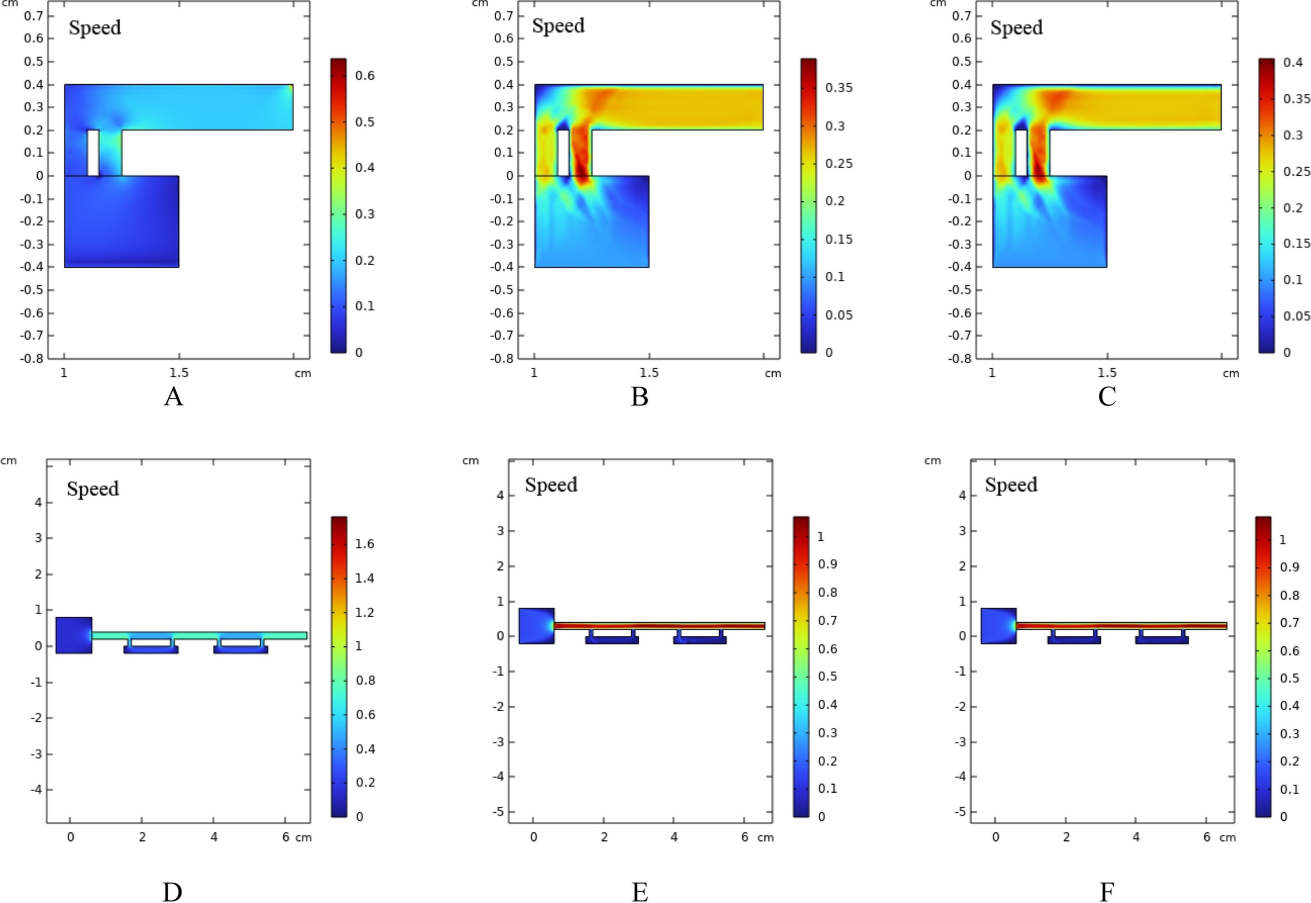
(A~C) Simulation diagrams of sweat flow velocity at time 0, 0.5, 1 in entrance section (D~F) Simulation diagrams of sweat flow velocity at time 0, 0.5, 1 in passageway section.

Capillary pumps are composed of multiple microstructure arrays that form narrow channels. When the fluid enters the capillary pump, the narrowness of the channels and the design of the microstructures provide the fluid with enhanced driving force.^[21]^ This increased driving force enables the capillary pump to maintain the spontaneous flow of the fluid and control the flow rate in a stable manner. (As shown in the Figure 5.)

### Analysis chambers

In the reaction chamber of a microfluidic device, two electrodes are typically placed for electrochemical analysis. Electrochemical analysis is an analytical technique based on electrochemical principles, which involves introducing appropriate potentials and currents to the electrode surface to detect electrochemical reactions in the sample and obtain analytical information.^[22]^

The reaction chamber contains modified electrodes for glucose and potassium ion detection. The working electrode is the electrode where the electrochemical reaction takes place. It directly contacts the sample under analysis, and by applying appropriate potentials and currents to its surface, the resulting current or potential changes from the electrochemical reaction are observed and recorded. In electrochemical analysis, the reference electrode provides a stable potential reference to ensure the accuracy and reproducibility of measurement results.

The potential difference between the working electrode and the reference electrode triggers the electrochemical reaction and generates the corresponding current or potential response. By controlling the applied conditions of potential and current, the monitoring and analysis of electrochemical reaction processes in the sample under analysis can be achieved.

The compact structure of the electrochemical reaction chamber in the microfluidic device, with a small distance between the electrodes, effectively reduces the diffusion distance of electrolytes, improving the efficiency and sensitivity of electrochemical reactions. Additionally, microfluidic technology enables microscale and high‐throughput analysis of samples, reducing reagent and sample waste while enhancing analysis efficiency.

By incorporating two electrodes in a microfluidic device and controlling the appropriate potentials and currents, electrochemical analysis can be achieved on a small scale, providing a convenient and efficient method for characterizing the electrochemical properties of the analyte. The integration of electrochemical analysis with microfluidic technology offers a new approach for rapid, sensitive, and high‐throughput electrochemical analysis, facilitating fast and efficient electrochemical analysis and detection in various fields.^[23]^

### Detection circuit

Connecting the designed electrochemical detection system to two detection electrodes, chronoamperometry and open circuit potential methods were employed for the electrochemical analysis of modified electrodes.^[24]^

**Figure 6:**
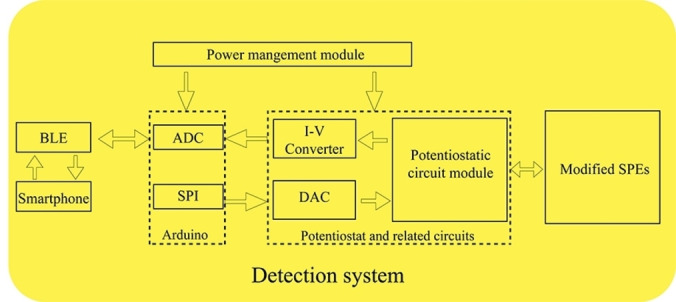
Complete Detection Circuit Design.

As shown in Figure 6, the hardware design utilized a commercial Arduino board with an analog‐to‐digital converter (ADC) as the microcontroller. In terms of application program design, the chronoamperometry and open circuit potential methods were integrated and developed for detection.^[25]^ The complete detection circuit primarily consisted of a microcontroller module, D/A module, potentiostat circuit module, Bluetooth module, and power management module.

The microcontroller module communicated with the external digital‐to‐analog converter via the Serial Peripheral Interface (SPI) to apply specific analog potential signals to the external screen‐printed electrodes (SPEs) for analysis. The potentiostat circuit module, integral to the detection system, comprises three Microchip MCP6072‐E/SN amplifiers. Functioning as a critical component, this module is designed to sustain a stable output between the working and reference electrodes, automatically compensating for potential drift during electrochemical reactions. Its role is pivotal in ensuring precise and reliable measurements, and the amplifiers within the module play a key role in amplifying and controlling electrical signals between electrodes, contributing to the overall accuracy of the electrochemical analysis in the system. The microcurrent detection circuit, including current‐to‐voltage conversion and amplification circuits, converted the reaction‐generated loop current between the working electrode and the counter electrode into an analog potential signal. This signal was then acquired and stored as a digital signal using the analog‐to‐digital converter (ADC) on the microcontroller.^[26]^

The collected electrical signals were transmitted via a low‐power Bluetooth module to a smartphone, which communicated with the detection circuit. On one hand, the smartphone sent instructions to the detection smartphone for selecting the detection method and setting initial parameters. On the other hand, it received and processed the digital signals from the detection circuit. Additionally, a computer, along with a button installed on the detection circuit, could also set the detection method and parameters through precompiled code.

The entire detection circuit was externally powered by a USB power source or dry battery pack, ensuring continuous and effective power supply through the power managementp2 module. It was used to send commands with initial parameters, such as voltage range, scan rate, pulse width, pulse period, and pulse amplitude. Based on the data, the electrical signals were calculated and signal curves were plotted for real‐time display on the smartphone.

## 2 Results and Discussion

### 2.1 Experimentation

In our study, we utilized indoor cycling as the mode of exercise to induce sweating. This choice was deliberate to ensure a controlled and consistent movement of the arms, facilitating the precise collection of sweat. we strategically placed the sensors on the participants′ arms to capture sweat generated during physical activity. This choice was made considering the arm‘s accessibility, participant comfort, and the physiological relevance of this location. The ease of operation and broad contact area on the arm were key factors, ensuring practicality and participant comfort. Additionally, the arm is known to produce relatively more sweat during movement, aligning with our focus on specific biomarkers during exercise. This placement directly relates to our research question, optimizing data collection for accurate and pertinent results. The decision to position the sensors on the arm reflects a thoughtful consideration of both practicality and physiological relevance, enhancing the overall validity and applicability of our study.

**Figure 7:**
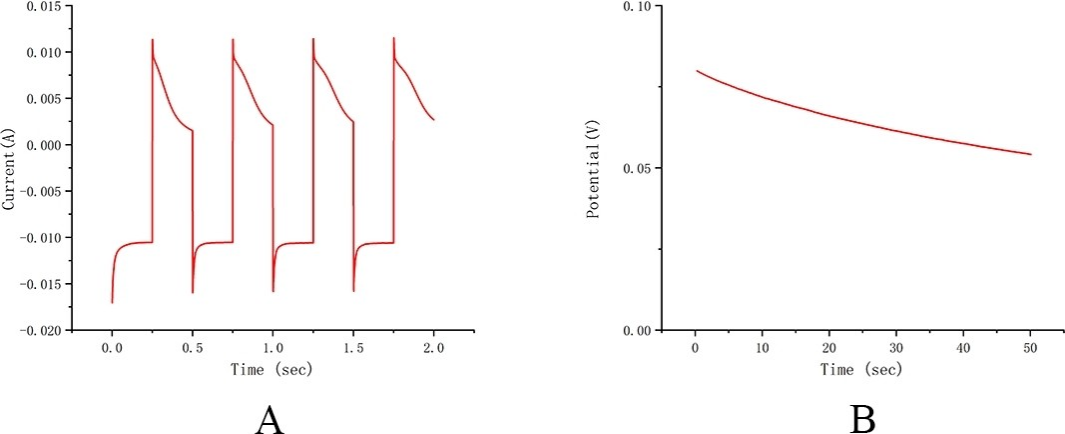
(A) Scanning glucose using an electrochemical workstation (B) Scanning K ions using an electrochemical workstation.

We used a professional electrochemical workstation to detect sweat samples after 20 minutes of exercise, as shown in the Figure 7. Used to compare with the equipment we have developed.

### 2.2 Blood glucose electrodes

#### 2.2.1 Retouching

Glucose oxidase (GOx) is highly selective and sensitive, and enzymatic glucose biosensors are considered ideal for wearable sensors.^[27]^

#### 2.2.2 Test data

**Figure 8:**
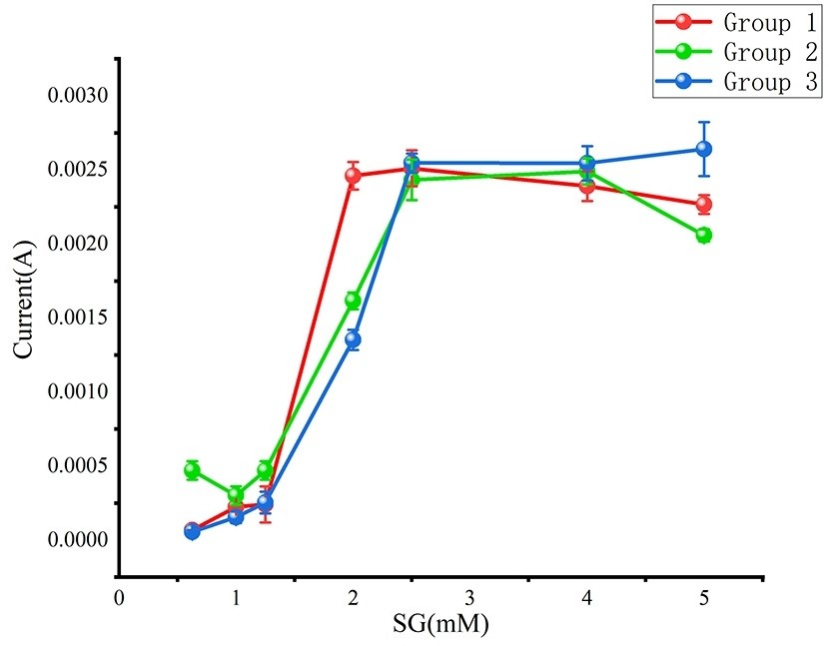
Relationship diagram between glucose concentration and current.

As shown in Figure 8, we conducted tests on seven concentrations of 0.625, 1, 1.25, 2, 2.5, 4, and 5 mM using an electrochemical workstation. Three sets of experiments were conducted for each concentration, and the relationship between glucose concentration and current was calculated based on the test results.

**Figure 9:**
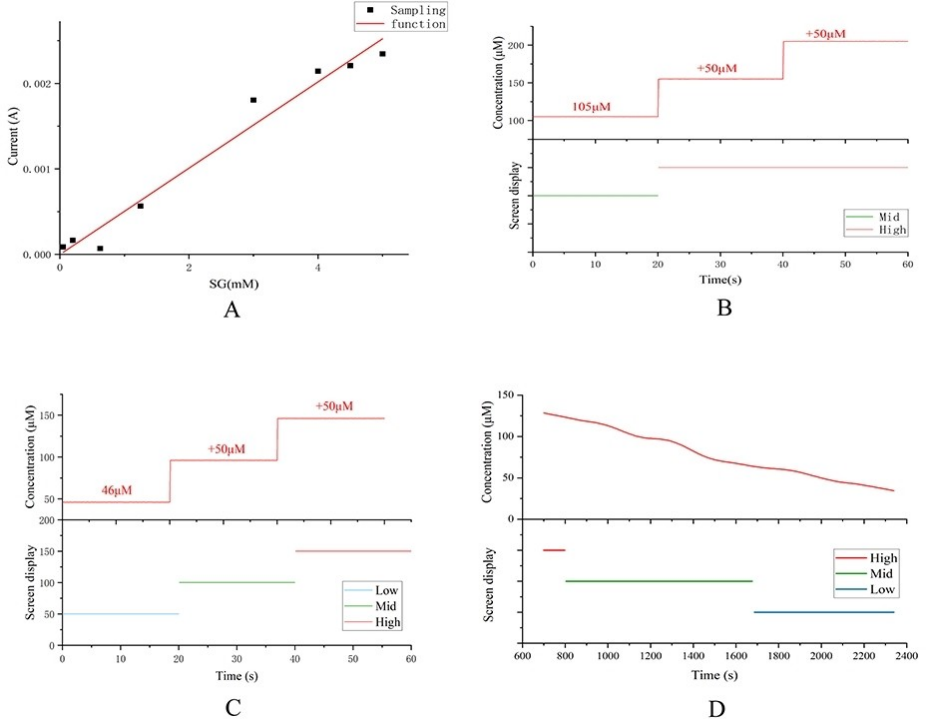
(A) Detection of different concentrations of glucose standard solutions with the sensor, generating a linear plot with a specific slope and intercept value. (B) (C) Validation of the accuracy of the sensor for monitoring sweat glucose levels based on sweat samples collected at different time points during exercise and sequentially increasing in concentration. The displayed sweat glucose levels (bottom) match well with the change in sweat glucose concentration (top). (D) Real‐time measurement of subjects′ sweat glucose levels during exercise.

We prepared glucose solutions with seven different concentrations of 0.625, 1, 1.25, 2, 2.5, 4, and 5 mM. By using the Chronoamperometry with an electrochemical workstation,^[28]^ making0 V as the initial potential, different concentrations of glucose standard solutions were scanned from 0 to 0.7 V. We conducted three sets of experiments at each concentration, resulting in the calibration curve shown in Figure 9.A. Based on this curve, the concentration can be calculated from the current. The Pearson correlation coefficient of 0.98 indicates highly accurate monitoring of blood glucose levels by the wearable sensor.

### 2.3 Hypoglycaemia prediction

Maintaining glucose levels within normal limits is essential for health management, and both high and low glucose levels can indicate the risk of metabolic disorders such as diabetes or hypoglycaemic shock, especially after strenuous exercise.[^29^, ^30^] To enable easy analysis of sweat glucose levels by the user, the output signal from the sensor is sent to the mobile phone via the Bluetooth module and the mobile app displays the “low”, “medium” and “high ” signals. Glucose concentrations of 60 μM and 120 μM were set as thresholds for the alarm signals with reference to relevant reports of sweat glucose monitoring. Figure 9.B, C shows a representative current response of the glucose sensor and the corresponding alarm signal on the mobile app. The glucose concentration in the sweat sample collected at 15 minutes during exercise was calculated from the current versus concentration curve in Figure 9.A to be 105 μM, at which point the mobile app shows medium sweat glucose level (“medium”). Adding glucose solution to the sample increased the concentration by 50 μM in turn, and the mobile app showed high sweat glucose levels (“high”). After 32 minutes of exercise, the glucose concentration in the sweat sample collected as shown in Figure. 9.C decreased to 46 μM and the mobile app showed “low” accordingly, again adding glucose solution to the sample to increase the concentration by 50 μM in sequence, the mobile app showed medium sweat glucose levels (“medium”) and high sweat glucose levels (“high”). The mobile app showed medium sweat glucose levels (“medium”) and high sweat glucose levels (“high”). This indicates that the sensor meets the design requirements. Figure 9.D. depicts the real‐time sweat glucose distribution over exercise time, a good demonstration of the reliability of monitoring blood glucose levels using wearable sensors.

### 2.4 Potassium ion electrodes

#### 2.4.1 Retouching

Valinomycin is a K^+^ selective ion carrier which can form complexes with K^+^ ions and, when modified to the electrode surface by a permeable membrane solution, can selectively recognize and permeate K^+^ ions, thus constructing a K^+^ selective electrode.^[31]^

#### 2.4.2 Test data

**Figure 10:**
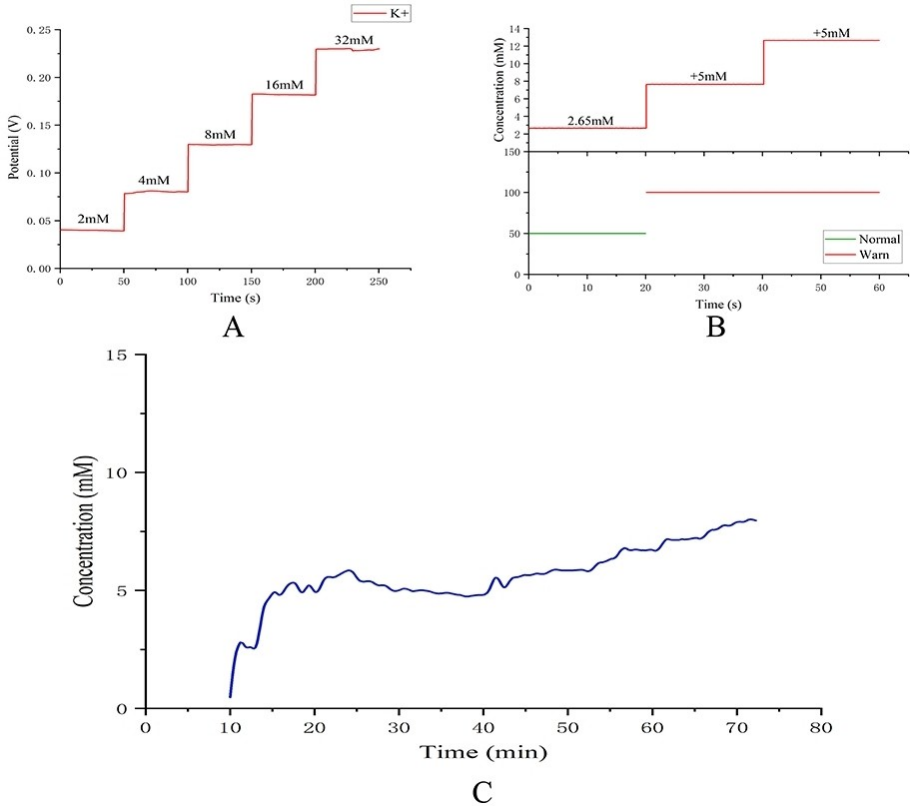
(A) Open circuit potential response curve of the sensor measuring K^+^ solution. (B) Sensor detection of sweat K^+^ to verify the accuracy of the sensor‘s sweat K^+^ level monitoring.(C) Real‐time measurement of sweat K^+^ levels in subjects during exercise.

The K^+^ testing is carried out in deionized water. For the tests, an open circuit potential was recorded for the process by using an open circuit potential method through the sensor to detect a concentration gradient of 2 mM to 32 mM K^+^ solution from −1 to 1 V.[^32^, ^33^] The test results are shown in Figure 10.A. the open circuit potential of the ion electrode increases proportionally with increasing concentration. The range of detection was from 2 mM to 32 mM. This range of concentrations tested covers the range of physiological variation in K^+^ concentration in sweat.

### 2.5 Water scarcity forecast

In order to enable easy analysis of sweat K^+^ levels by the user, the output signal from the sensor is sent to the mobile phone via the Bluetooth module and the mobile phone app displays the signals “Normal” and “Warn”. A K^+^ concentration of 7.5 mM was set as the threshold for the alarm signal, taking into account the relevant sweat K^+^ monitoring reports. Based on the sweat K^+^ level of the subject during exercise in Figure 10.C the K^+^ concentration in the sweat sample collected at 10–15 minutes during exercise was measured to be 2.65 mM, as shown in Figure 10.B when the mobile app displayed “Normal”. Adding K^+^ solution to the sample causes the concentration to increase by 5 mM in turn and the mobile app displays “Warn”. The wearable sensor is monitoring the K^+^ concentration as expected.

## 3 Conclusions

This paper effectively utilizes microfluidic technology to collect sweat and convert it into quantifiable droplets for monitoring and analysis. By integrating microfluidic channels with glucose and K+ sensors, a device has been developed to remind users of the risks of low blood glucose and dehydration during home exercise routines. In the future development, it is important to consider additional biochemical markers: Apart from glucose and potassium ions, sweat contains other important biochemical markers such as lactate, sodium ions, and uric acid. The next steps of development can focus on detecting these markers and investigating their relationships with health conditions and diseases. This will contribute to the establishment of a comprehensive sweat biochemical analysis platform, providing valuable information for personalized healthcare.

## Conflict of interests

The authors declare no conflict of interest.

4

## Data Availability

The data that support the findings of this study are available on request from the corresponding author. The data are not publicly available due to privacy or ethical restrictions.
